# Mosquito biodiversity and mosquito-borne viruses in the United Arab Emirates

**DOI:** 10.1186/s13071-019-3417-8

**Published:** 2019-04-03

**Authors:** Jeremy V. Camp, Noushad Karuvantevida, Houda Chouhna, Ebtesam Safi, Junid N. Shah, Norbert Nowotny

**Affiliations:** 1 Viral Zoonoses, Emerging and Vector-borne Infections Group, Institute of Virology, University of Veterinary Medicine Vienna, Vienna, Austria; 2 Department of Basic Medical Sciences, College of Medicine, Mohammed Bin Rashid University of Medicine and Health Sciences, Dubai, United Arab Emirates; 3 Natural Resources Conservation Section, Environment Department, Dubai Municipality, Dubai, United Arab Emirates

**Keywords:** Urbanization, Bagaza virus, Barkedji virus, Flavivirus

## Abstract

**Background:**

In the last 50 years, the United Arab Emirates (UAE) has experienced rapid population growth and urbanization. Urbanization is known to influence biodiversity, and there appears to be a link between the emergence of arboviruses and urban growth. Very little is known about the UAE mosquito species richness and dominant vectors. We performed a mosquito survey comparing peri-urban sites in Dubai and Al Ain to a protected, natural site in Fujairah emirate. We measured mosquito biodiversity and species composition, and screened mosquito pools for common arboviruses to measure arbovirus activity in the region.

**Results:**

We report ten species of mosquitoes from the UAE, with highest species diversity in the natural site, a protected wadi near the eastern coast. The predominant mosquito was *Culex perexiguus*, and was associated with peri-urban habitats. The site with lowest mosquito species diversity but relatively high species richness was the peri-urban site of Al Ain Zoo, where we identified Bagaza virus and Barkedji virus, two flaviviruses, in pools of *Cx. perexiguus*.

**Conclusions:**

Decreased mosquito biodiversity was associated with increased levels of urbanization. The predominance of two species at peri-urban sites was related to the availability of their larval habitats. Arboviruses were associated with the presence of a single predominant mosquito species, *Cx. perexiguus*.

## Background

The United Arab Emirates (UAE) has experienced an extremely high rate of urbanization and development in the last 50 years. Urban centers of Dubai and Al Ain (eastern Abu Dhabi emirate) have experienced rapid urban sprawl, with increases in tourism, shipping, and the population of foreign laborers [1, 2]. Between 1972 and 2011, urban land cover in Dubai emirate increased at an annual growth rate of 10%, with a peak growth rate of 13% between 2003–2005, likely making Dubai the fastest growing city in the world during the first decade of the 21st century with 15% of total land area classified as urban areas in 2011 [1]. Urban land cover has grown at a similarly high rate in Al Ain municipality, with an estimated 26.2% of the total area classified as urban area [3]. Urbanization and anthropogenic landscape alteration have major effects on biodiversity [4, 5]. Importantly, urbanization may also affect the biodiversity and abundance of disease vectors, which in turn may influence the emergence of vector-borne diseases [6, 7].

It has been estimated that over 20% of emerging infectious diseases are vector-borne [8], with several (re-)emerging mosquito-borne arboviruses causing global epidemics in the last 20 years. Arbovirus emergence is linked to urbanization in many ways [8–10]. Urban sprawl and expanding agriculture increase the probability of spillover of enzootic arbovirus transmission cycles into humans and livestock [11]. Tourism, animal trade, and human migration from countries with autochthonous arbovirus transmission may further provide a means for arbovirus introduction and emergence [12]. Urban centers and peri-urban areas are sites of high population density, and pose epidemiological challenges for the control of outbreaks [8, 10]. Global trade and transportation have resulted in the introduction of arbovirus vectors and subsequent adaptation of vectors to urban areas resulting in increased contact rate between vectors and hosts, e.g. *Aedes* (*Stegomyia*) *aegypti* L. [9, 13]. Furthermore, urbanization likely affects mosquito biodiversity and abundance, and may influence trophic interactions between mosquitoes and their hosts in complex ways [6, 7, 14, 15]. Despite the rapid rate of urbanization and anthropogenic habitat alteration, very little is known about the biodiversity of potential arbovirus vectors and arbovirus activity in the UAE.

There is some evidence of autochtonous transmission of arboviruses in the UAE and surrounding area. West Nile virus (*Flaviviridae*, *Flavivirus*) was isolated for the first time in the UAE from a dromedary camel in 2016 [16]. In a recent study in Oman, we identified Sindbis virus (*Togaviridae*, *Alphavirus*) and Barkedji virus (BJV) (*Flaviviridae*, *Flavivirus*) in a local *Culex* (*Culex*) *quinquefasciatus* Say population (N. Nowotny, unpublished data). Whether BJV infects vertebrate hosts has yet to been determined [17]. More is known about the mosquito-borne viruses present in neighboring countries of the Arabian Peninsula, most notably the introduction of Rift Valley fever virus (RVFV) (*Phenuiviridae*, *Phlebovirus*) in Saudi Arabia and Yemen in 2000 [18, 19]. The Rift Valley fever outbreak resulted in 884 hospitalized patients in Saudi Arabia with 124 deaths, and 1087 cases with 121 deaths in Yemen [20]. Dengue virus (*Flaviviridae*, *Flavivirus*) is also present in these two countries, and has caused recent outbreaks [21–24]. Autochthonous cases of chikungunya virus (*Togaviridae*, *Alphavirus*) have also been reported from the Arabian Peninsula, again from neighboring Saudi Arabia [25] and Yemen [21], but not yet from the UAE. Zika virus (*Flaviviridae*, *Flavivirus*) cases have not yet been reported from the Middle East [22]; however, Zika virus is present in the Maldives and other vacation destinations of those living in the UAE [26]. Many of these arboviruses may cause mild and/or subclinical symptoms in infected human patients, and to our knowledge, no serological or virological surveys of humans have been performed in the UAE.

Despite these reports, a detailed survey of mosquito biodiversity focusing on the UAE has never been performed, nor has arbovirus surveillance within the mosquito population been performed. The little information available on the mosquito species present in the UAE is limited to single species accounts [27, 28]. The mosquito populations of neighboring Saudi Arabia and Iran, across the Arabian/Persian Gulf, are better described [29–33]. Recent urbanization has likely created additional habitats for urban-adapted mosquito species, and increased man-made agricultural works which seek to ‘make the desert green’ provide many man-made bodies of water for the development of immature mosquitoes. We performed entomological surveys to measure mosquito biodiversity in the UAE, and screened the resulting mosquito pools for the presence of arbovirus nucleic acids. Specifically, we tested the hypothesis that mosquito biodiversity and arbovirus prevalence may be influenced by urbanization by sampling in three habitats: two peri-urban green spaces, and one natural habitat.

## Methods

### Study sites

**Fig. 1:**
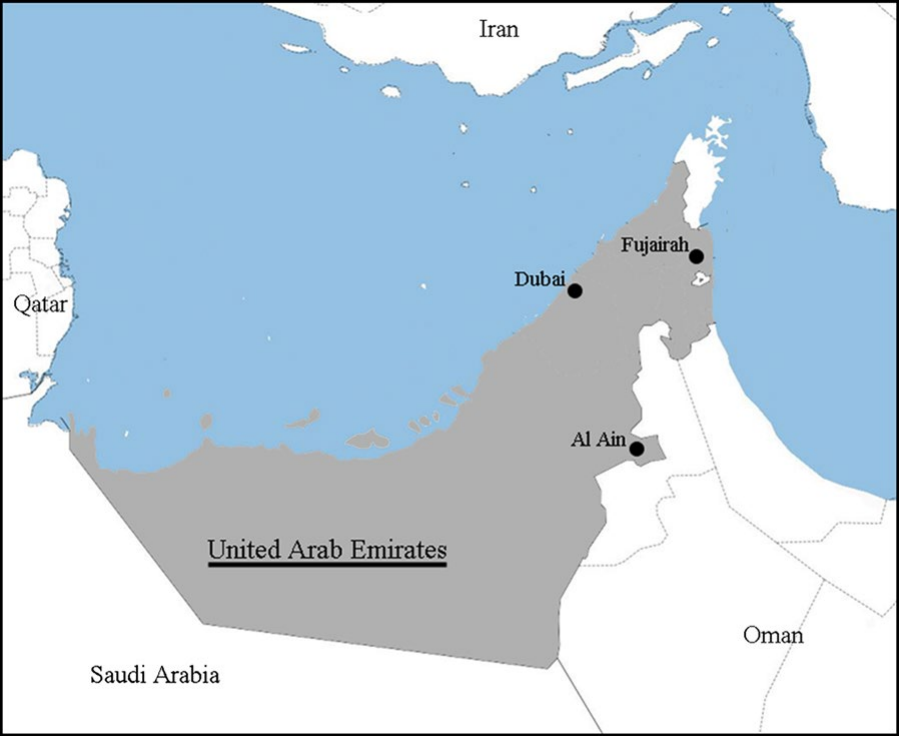
Mosquito sampling locations in the United Arab Emirates: Dubai (2 sites), Al Ain (3 sites), and Wadi Wurayah in Fujairah emirate (black points plotted on background map, labels and shading added: ©OpenStreetMap contributors, CC BY-SA)

Three principal locations were chosen to place traps: disturbed artificial wetlands in the highly urbanized Emirate of Dubai (25°11.334′N, 55°18.814′E), peri-urban areas in Al Ain (24°10.696′N, 55°44.358′E), and a protected natural habitat in Wadi Wurayah National Park (WW) in the Emirate of Fujairah (25°23.787′N, 56°16.176′E) (Table 1, Fig. 1). Two trapping sessions were conducted: winter (from 30 January 2018 until 11 February 2018) and spring (from 16 April 2018 until 2 May 2018). The winter trapping session sampled multiple habitats in each location, whereas the spring trapping session focused on specific sites within a location which were identified during the winter as having relatively high mosquito abundance.

**Table 1:** Mosquito sampling effort in the United Arab Emirates during Winter (January–February) and Spring (April–May) trapping seasons, 2018

Trapping location	Coordinates	Trap-nights (LT-BG-BL)^a^	
		Winter	Spring
Al Ain, Oasis	24.218°N, 55.768°E	1–1–1	0–0–0
Al Ain, Lake Zakher	24.175°N, 55.627°E	1–0–1	0–0–0
Al Ain, Zoo (AAZ)	24.175°N, 55.740°E	4–1–2	3–2–0
Dubai, Ras al Khor (RK)	25.186°N, 55.330°E	2–2–1	2–5–2
Dubai, Qudra Lakes	24.841°N, 55.368°E	1–0–0	0–0–0
Fujairah, Wadi Wuraya (WW)	25.396°N, 56.269°E	2–2–1	4–3–0

^a^Numbers indicate nights with light traps baited with dry ice (LT), nights with BG sentinel traps baited with dry ice (BG), as well as BG sentinel traps baited with BG Lure (“BL”, a commercial preparation of caproic acid, lactic acid and ammonia), separated by an en dash

Habitat descriptions are based on our observations and habitat classifications under the Ramsar wetland classification system [34]. In Dubai, traps were placed at Ras Al Khor Wildlife Sanctuary (RK) and Al Qudra Lakes during the winter trapping session and only at RK during the spring session (Table 1). RK is a 620 ha wildlife sanctuary at the interface between the Arabian Gulf and Al Awir Desert, 5 km southeast of city center and enclosed by the highly urbanized city of Dubai. It is a tidal, coastal, semi-natural wetland consisting of brackish water fed by treated municipal wastewater mixing with seawater from Dubai Creek. The dominant vegetation type is an extensive mangrove, *Avicennia marina* (Forssk.), swamp which was planted from 1991–1994. The Ramsar Wetland classification scheme lists it as containing the following wetland types: intertidal marsh; intertidal mud, sand and salt flats; intertidal forested wetlands (mangrove swamp); and man-made artificial lagoon. In Al Ain, winter trapping was performed in three sites, the Al Ain oasis, Lake Zakher and Al Ain Zoo (AAZ), and spring trapping was performed only at AAZ (Table 1). The AAZ is a man-made landscaped habitat, which houses over 4000 animals from 180 species on a 400 ha plot with diverse plant species. Water sources include artificial concrete ponds and streams, and numerous artificial containers. Land cover/land use mapping has classified it as a leisure area surrounded by low-density urban, disturbed ground, and industrial land cover. The protected area of WW is approximately 22,400 ha within the Arabian highlands and shrublands ecoregion (WWF Global 200 Ecoregion 127), which includes inland perennial freshwater habitats such as streams, waterfalls and rock pools. It is classified under the following wetland types by the Ramsar wetland classification scheme: permanent streams; seasonal/intermittent/irregular streams; permanent freshwater marshes/pools and ponds below 8 ha; seasonal/intermittent freshwater marshes/pools below 8 ha; freshwater springs and oases. At WW, trap stations were moved nightly within a 0.5 km stretch along an ephemeral riverbed near waterfalls and rock pools (Table 1).

### Trapping methods

Sites were briefly surveyed by 15 min landing count at dusk and visual inspection of aquatic sites for immature mosquito stages. Once potential sites were identified, adult females were sampled using two trap designs: BG Sentinel trap (BGS) (BioGents, Regensburg, Germany) and a CDC miniature light trap (LT) (J.W. Hock, Gainseville, FL, USA). Both trap types were run overnight (from 1 h before sunset to 1 h after sunrise) and baited with CO_2_ (0.5 kg dry ice). In addition, to target anthropophilic mosquito species, BG Lure, a commercial preparation of ammonia, lactic acid, and caproic acid delivered on saturated granules (BioGents, Regensburg, Germany), was used as an attractant in a BGS for one trap-night per site. To compare trapping efficiencies, the sampling effort was designed to be equal at each location for each season; however, technical difficulties were encountered. Therefore statistical analyses are performed only for LT or BGS using CO_2_, and collections are adjusted by trap-night per season (Table 1).

### Mosquito identification

Trap contents were transferred to -80 °C until identification. Mosquitoes were sorted on dry ice using a stereoscopic microscope, pooled by species in pools < 30 individuals, and returned to storage at -80 °C. Species identifications were based on morphological characters described in publications which catalog the mosquito fauna of Iran, Egypt and southwest Asia [31, 33, 35, 36]. Single voucher specimens from each morphological species identification per site were photographed and then processed for molecular barcoding. DNA was extracted from each voucher specimen using a commercial kit (DNeasy, Qiagen, Hilden, Germany) and PCR was used to amplify a 648 bp portion of the cytochrome *c* oxidase subunit 1 (*cox*1) gene using primers VF1d and VR1d as described in [37]. Amplicons were sequenced by Sanger method (Microsynth AG, Vienna, Austria), and sequences were compared to GenBank sequence database using nBlast search (http://blast.ncbi.nlm.nih.gov). Voucher sequences were deposited in the GenBank database under the accession numbers MK170082-MK170098. Specific morphological identification could not be performed on some damaged specimens and are excluded from analysis of mosquito biodiversity. Mosquito biodiversity at each of the main trap sites (urban, peri-urban, natural) was measured by calculating species richness (*S*_*obs*_) and the Chao 2 estimate for species richness based on replicated incidence data (*S*_*Chao2*_) [38] (which has previously been used to estimate mosquito species richness [39]); alpha diversity using Shannon index (*H*′ =  -Σ *p*_i_ ln *p*_*i*_, where *p*_*i*_ is the proportion of species *i*, summed for all species at the site); and evenness (*J*′ = *H*′/ln *S*). Trapping efficiency (i.e. the number of mosquitoes per trap-night) was used as a measure of mosquito abundance and statistical comparisons of trapping efficiency used exact binomial tests under the null hypothesis of equal distribution (i.e. between traps or between seasons) with type I error set to *α* = 0.05.

### Arbovirus screening

Mosquito pools were homogenized in buffer on a bead mill (PowerLyzer 24, Qiagen) and homogenates were cleared by centrifugation. RNA was extracted from 140 µl of the resulting supernatant using a commercial kit (QIAamp viral RNA mini kit, Qiagen) and stored at -80 °C until analysis. Alphavirus RNA detection followed a published nested protocol [40] wherein 1 µl of first-round RT-PCR product is used in a second PCR reaction. Additionally, samples were tested for chikungunya virus, specifically, using a published probe-based RT-qPCR protocol [41] and a commercial kit (Luna probe RT-qPCR, New England Biolabs, Ipswich, MA USA). Similarly, RVFV was detected by RT-qPCR using a published protocol [42]. Samples were tested for orthobunyaviruses (*Peribunyaviridae*) by RT-PCR using three published protocols targeting California and Bunyamwera group viruses [43], California and Bwamba group viruses [44], and Bunyamwera group viruses [44]. Flavivirus RNA was detected by RT-PCR using MAMD and cFD2 primers as previously described [17, 45, 46]. As flavivirus screening revealed some pools which potentially contained more than one flavivirus, a BJV-specific RT-PCR assay was designed using the primers BJV_F (5′-AAT ACG GAG CGG GAA CAC-3′) and BJV_R (5′-CTG GAT GAC ACT CCT TTC AT-3′). The BJV-specific RT-PCR was performed with a commercial kit (One*Taq* one-step RT-PCR, New England Biolabs), and thermocycler conditions were as follows: 30 m incubation at 50 °C followed by 1 m incubation at 94 °C; then a touch-down PCR procedure was used by annealing at 60 °C and then decreasing 1 °C for each cycle for 10 cycles, followed by 35 cycles at 50 °C annealing temperature for 30 s, where melting and extension steps were performed at 94 °C and 68 °C, respectively, for 30 s each cycle; and a final extension step at 68 °C for 5 m. A similar RT-PCR protocol was designed for Bagaza virus (BAGV); however, this protocol was not successfully optimized. All RT-PCR reactions were visualized with GelRed dye following gel electrophoresis on a 1.2% agarose gel. Amplicons of a predicted size were cut from the gel, the DNA was cleaned with a commercial kit (Wizard SV, Promega, Madison, WI, USA), and the samples were sequenced by Sanger method and compared to published sequences in GenBank using nBlast. Virus sequences were deposited in the GenBank database under the accession numbers MK170099-MK170104.

## Results

### Mosquito collections

A total of 1142 mosquitoes were collected from the three main collection stations (AAZ, RK, WW). Mosquito abundance was lower at secondary sites at Qudra lakes (near Dubai) and Lake Zakher (near Al Ain), only collecting 2 specimens each in the winter season, and therefore collections were not continued at these sites. Sampling at Al Ain oasis produced a single male anopheline mosquito which was not identified to species, and it was later discovered that insecticides were being used to control pests of the date palms at that site. Trap-nights using BGS with BG Lure captured no mosquitoes. Mosquitoes at WW were less abundant than at other collection stations: 7.0% of total collections were from WW; 53.6% of total collections were from AAZ; and 39.0% of total collections were from RK (the remaining 0.4% were from Qudra lakes and Lake Zakher).

DNA barcoding of voucher specimens confirmed morphological identifications, and it was found that 10 species were represented (Table 2). *Culex* (*Culex*) *perexiguus* Theobald was the predominant species collected across all locations (*n* = 710), and was the most common mosquito at AAZ (*n* = 549; 89.7% of total collections at AAZ), though made up only 33.3% (*n* = 148) of collections at RK and 12.9% (*n* = 11) of collections at WW. *Aedes* (*Ochlerotatus*) *caspius* (Pallas) (*n* = 287) was the predominant species collected at RK (*n* = 279; 62.7%), but made up only 1.3% of collections at AAZ (*n* = 8) and was not captured at WW. *Culex quinquefasciatus* (*n* = 82) comprised 50.6% of the total collection at WW (*n* = 43), but was less frequently encountered at AAZ (*n* = 31; 5.1%) and RK (*n* = 8; 1.8%). The collections at WW included three species not encountered elsewhere: *Cx.* (*Oculeomyia*) *bitaeniorhynchus* Giles (*n* = 5); *Cx.* (*Cx.*) *laticinctus* Edwards (*n* = 2); and *Cx.* (*Cx.*) *sinaiticus* Kirkpatrick (*n* = 13). Three species were unique to AAZ: *Anopheles* (*Cellia*) *culicifacies* Giles (*n* = 10); *An.* (*Cel.*) *stephensi* Liston (*n* = 1); and *Cx.* (*Cx.*) *tritaeniorhynchus* Giles (*n* = 8). Two *Cx.* (*Cx.*) *sitiens* Wiedmann were captured from the winter trap site at Lake Zakher and nowhere else. Finally, *Culiseta* (*Allotheobaldia*) *longiareolata* (Macquart) was encountered at a low relative frequency at AAZ (*n* = 2) and WW (*n* = 6).

**Table 2:** Mosquito (Diptera: Culicidae) species abundance using two trap designs baited with CO_2_ at three locations in the United Arab Emirates, 2018

Species	Al Ain^a^			Dubai^b^			Fujairah^c^			Grand total
	BG^d^	LT^d^	Total	BG	LT	Total	BG	LT	Total	
*Aedes caspius*	5	3	8	229	50	279	0	0	0	287
*Anopheles culicifacies*	9	1	10	0	0	0	0	0	0	10
*Anopheles stephensi*	1	0	1	0	0	0	0	0	0	1
*Culex bitaeniorhynchus*	0	0	0	0	0	0	3	2	5	5
*Culex laticinctus*	0	0	0	0	0	0	1	1	2	2
*Culex perexiguus*	102	447	549	69	81	150	11	0	11	710
*Culex quinquefasciatus*	10	21	31	6	2	8	40	3	43	82
*Culex sinaiticus*	0	0	0	0	0	0	13	0	13	13
*Culex sitiens*	0	2	2	0	0	0	0	0	0	2
*Culex tritaeniorhynchus*	1	7	8	0	0	0	0	0	0	8
*Culiseta longiareolata*	2	0	2	0	0	0	5	1	6	8
Grand total	130	481	611	304	133	437	73	7	80	1128

*Note*: Specific sites at each location are given in Table 1

^a^Sampling in Al Ain was performed at Al Ain Zoo, Al Ain oasis and Lake Zakher

^b^Sampling in Dubai was performed at Ras Al Khor and Al Qudra lakes

^c^Sampling in Fujairah emirate was performed at national park Wadi Wurayah

^d^Traps included BG sentinel traps (BG) or light traps (LT) baited with CO_2_ in the form of dry ice

Some species showed seasonality in their abundance: *Cx. sinaiticus* (binomial test of equal distribution, *P* < 0.001), and *Cx. tritaeniorhynchus* (*P* = 0.007) were only captured in the winter, and 81 *Cx. quinquefasciatus* were captured in the winter and only 1 captured in the spring (*P* < 0.001). Significantly more *Ae. caspius* (*P* < 0.001), *An. culicifacies* (*P* = 0.02), and *Cx. perexiguus* (*P* < 0.001) were captured in the spring, although all three species were also captured in the winter season.

Trapping efficiency was highest at AAZ, where LT caught 116.6 mosquitoes per trap-night over 5 trap-nights (BGS captured 14 mosquitoes over 1 trap-night). BGS and LT traps performed at similar collection efficiencies at RK with 44.6 and 42.3 mosquitoes per trap-night, respectively (BGS, 7 trap-nights; LT, 3 trap-nights); and WW had the lowest collection efficiency with BGS traps collecting 8.0 mosquitoes per trap-night and LT collecting 3.8 mosquitoes per trap-night over 5 trap-nights each. Two species showed clear trap bias (*P* < 0.01): *Ae. caspius* was captured at a 4.5-fold higher rate per trap-night in BGS traps than in LT (17.6 and 3.9 per trap-night, respectively); and *Cx. perexiguus* was captured at a 7.2-fold higher rate per trap-night in LT than in BGS (47.3 and 6.5 per trap-night, respectively). All other species were captured at a rate of less than 1 per trap-night, with the exception of *Cx. quinquefasciatus* (average rate of 2.5 per trap-night).

Comparing the species richness estimator (*S*_*Chao2*_) to the observed species richness (*S*_*obs*_) suggested that sampling at WW (*S*_*obs*_ = 6; *S*_*Chao2*_ = 6.0; 95% CI: 6.72–7.24) and RK (*S*_*obs*_ = 3; *S*_*Chao2*_ = 3.0; 95% CI: 3.0–3.3) was comprehensive, but further sampling at AAZ would likely reveal more mosquito species (*S*_*obs*_ = 8; *S*_*Chao2*_ = 9.9; 95% CI: 8.15–19.7) (Table 3). Species richness was highest in the winter, with seven species encountered at AAZ (*S*_*Chao2*_ = 8.2; 95% CI: 7.1–17.8) and six species encountered at WW (*S*_*Chao2*_ = 15.4; 95% CI: 7.2–77.0), yet only three species were encountered at RK (*S*_*Chao2*_ = 3.0; 95% CI: 3.0–4.0) (Table 3). The highest species alpha diversity was from WW, *H*′ = 1.36 (*H*′_*winter*_ = 1.03; *H*′_*spring*_ = 1.02). Species diversity was lowest at AAZ (*H*′ = 0.46), with a higher species diversity in the winter than in the spring (*H*′_*winter*_ = 0.56; *H*′_*spring*_ = 0.24). RK had an intermediate species diversity (*H*′ = 0.73), with a comparatively high diversity in the winter (*H*′_*winter*_ = 0.92) and lower in the spring (*H*′_*spring*_ = 0.63). The high species diversity at WW also displayed higher evenness (*J*′ = 0.76), whereas other collection stations were dominated by a single species: *Cx. perexiguus* represented 89.7% of the collection at AAZ (*J*′ = 0.23) and *Ae. caspius* represented 63.8% of the total collection at RK (*J*′ = 0.66). The evenness at RK in the winter sampling season was the highest recorded from all sites (*J*′_*winter*_ = 0.84; *J*′_spring_ = 0.58). Evenness at AAZ was similarly higher in the winter than in the spring (*J*′_*winter*_ = 0.31; *J*′_spring_ = 0.17), and evenness at WW was higher in spring than in winter (*J*′_*winter*_ = 0.57; *J*′_spring_ = 0.74).

**Table 3:** Biodiversity estimates [Shannon diversity index (*H*′), evenness (*J*′ = *H*′*/*ln*(S)*), species richness (*S*_*obs*_), and Chao2 estimator of species richness (*S*_*Chao2*_) with 95% confidence intervals in parentheses] of the mosquito populations sampled from three regions in United Arab Emirates, 2018, during two trapping seasons using CDC light traps and BG sentinel traps baited with CO_2_

Site	Season	*H*′	*J*′	*S*_*obs*_	*S*_*Chao2*_
Al Ain Zoo	Winter	0.56	0.31	7	8.2 (7.1–17.8)
	Spring	0.24	0.17	4	6.2 (4.3–22.5)
	Total	0.46	0.23	8	9.3 (8.2–19.7)
Ras Al Khor	Winter	0.92	0.84	3	3.0 (3.0–4.0)
	Spring	0.63	0.58	3	3.0 (3.0–4.4)
	Total	0.73	0.66	3	3.0 (3.0–3.3)
Wadi Wurayah	Winter	1.03	0.57	6	15.4 (7.2–77.0)
	Spring	1.02	0.74	4	6.9 (5.2–21.6)
	Total	1.36	0.76	6	6.0 (6.0–7.2)
Grand total		1.04	0.45	10	12.4 (11.2–23.6)

### Arbovirus screening

Seventy-four pools of mosquitoes were tested for the presence of common arboviruses. All pools were negative for viral nucleic acids from the following virus taxa: orthobunyaviruses (California, Bunyamwera and Bwamba serogroups) and RVFV. Two pools of *Cx. perexiguus* mosquitoes from AAZ were putatively positive for chikungunya virus by RT-qPCR, however further testing with pan-alphavirus RT-PCR resulted in no product and therefore these were concluded to be false-positives. Fifteen pools were positive for flavivirus RNA using conventional pan-flavivirus RT-PCR. Sequencing the 15 RT-PCR products produced BJV sequences from 6 pools, 1 pool with BAGV sequence and 5 pools which appeared to be mixed BJV and BAGV infections based on inspection of sequencing chromatographs. Sequences obtained from the remaining three of 15 putative positive samples (one pool of *Cs. longiareolata* from AAZ and two pools of *Ae. caspius* from RK) were determined to be non-specific reactions (not a viral sequence) producing a product similar to the expected size. All 12 pools from which flavivirus sequence(s) were detected were made up of *Cx. perexiguus* captured on a single night with a single trap at AAZ, and many pools contained gravid individuals.

A virus-specific conventional RT-PCR was developed and used to amplify BJV from a pool that produced a clear BAGV sequence by universal flavivirus RT-PCR. Thus it was concluded that 11 pools of *Cx. perexiguus* were positive for BJV, at least 6 of those were positive for BAGV nucleic acid, and specific sequences of each virus were obtained from at least one of these pools. The 214 bp BAGV sequences from two pools were identical to each other (MK170099), and had 99.1% sequence identity to a sequence isolated from a pool of *Cx. quinquefasciatus* in Zambia in 2013 (LC318701). Five BJV-positive sequences (MK170100-MK170103, including one sequence isolated from a pool that was also BAGV-positive, MK170104), were 99.5% identical to each other, differing in one nucleotide over the 214 bp sequence, and a 299 bp sequence (MK170104) had 95% sequence identity to sequences isolated from *Cx. perexiguus* in Israel 2011 (KC496020) [16] and an isolate from Senegal (EU078325).

## Discussion

An extensive compilation of mosquito species in Arabia published in 1956 [47] lists the following species as found in “Trucial Oman”, which is now UAE: *Ae. aegypti*; *An. culicifacies*; *An.* (*Cel.*) *multicolor* Cambouliu; *An. stephensi*; *Cs. longiareolata*; *Cx. pipiens*; “*Culex fatigans*” (= *Cx. quinquefasciatus*); and *Cx. sitiens*. In a review of the subgenus *Culex* in southwestern Asia, Harbach [31] noted that *Cx. pipiens*, *Cx. quinquefasciatus* and *Cx. sitiens* were likely distributed in the UAE, but stated that too few species descriptions exist to make an accurate distribution record. Indeed, ten years later in 1998, Harbach [48] collected *Cx. sitiens*, *Cx. tritaeniorhynchus* and *Culex* (*Barraudius*) *pusillus* Macquart in Dubai municipality. In a catalog of anopheline mosquitoes of southwestern Asia and Egypt, Glick [35] reported *An. culicifacies*, *An.* (*Cel*.) *dthali* Patton, *An*. (*Cel*.) *paltrinieri* Shidrawi and Gillies, *An*. (*Cel.*) *sergentii* (Theobald) and *An*. *stephensi* from the UAE, noting that Akoh et al. [49] used cytogenetics to identify *An*. *culicifacies* species A from Al Ain. Finally, a recently published account of several *Aedes* spp. found in western Saudi Arabia listed several species not captured in our survey, including *Ae.* (*Aedimorphus*) *vexans arabiensis* Patton and *Ae. aegypti* [50]. The trapping methods used here are known to be effective collection methods for mosquitoes in the subgenus *Stegomyia*; however, none were collected [51, 52]. However, we acknowledge that we only used methods to sample adult mosquitoes and only sampled for two seasons in the year. The estimates of species richness suggest that further sampling, particularly in peri-urban habitats, would reveal more species.

With some exceptions (*Ae. aegypti*, *Ae. vexans arabiensis*, *An. dthali*, *An. paltrinieri*, *An. sergentii*, *An. multicolor*, *Cx. pipiens* and *Cx. pusillus*), we confirm the previously recorded species are still found in the UAE (Table 2). More recent reports from Saudi Arabia and Iran suggest that our reports of *Ae. caspius*, *Cx. bitaeniorhynchus*, *Cx. laticinctus*, *Cx. perexiguus* and *Cx. sinaiticus* are not surprising occurrences in the UAE, although they were not specifically included in earlier species accounts [29–33]. The anopheline species recorded in our survey, including *An. culicifacies* which were only found in Al Ain, are well-known primary malaria vectors in India and southwest Asia [53]. Extensive mosquito control regimes were enacted in the last 30 years targeting malaria vectors, and the UAE was declared “malaria free” in 2007 by the World Health Organization [54]. Eradication efforts may have contributed to the low abundance and low species diversity of the anopheline mosquitoes in our survey.

*Aedes caspius* was the only aedine species found in our survey, and was the most frequently collected species at RK. It is a widely distributed Palaearctic species which has a tolerance for waters with high salinity, and is typically found in inland salt marshes much like the habitat at RK [55]. It is a competent vector of RVFV [56]. Flaviviruses, including WNV, have previously been identified from pools of female *Ae. caspius* [57], although it is likely not an efficient vector of WNV [58].

*Culex perexiguus* was the predominant species in AAZ, and the second most frequently collect species at RK. It is a member of the Univitattus complex, and both *Cx.* (*Cux*.) *univitattus* Theobald and *Cx. perexiguus* are present on the Arabian Peninsula [31]. Although we did not inspect male terminalia to differentiate *Cx. univitattus* from *Cx. perexiguus*, the molecular bar-coding performed here and the distribution in the eastern Arabian Peninsula suggest that we captured *Cx. perexiguus* [31, 33, 59]. Based on the presence of WNV nucleic acid in mosquito pools and ornithophilic host preference, it has been suggested that the Univitattus complex, likely *Cx. univitattus* (*s.s.*), is an important enzootic vector of WNV in Portugal and Spain [59–63] as well as in Egypt and southwest Asia [31, 64]. It is known to be a competent vector of RVFV [56]. Herein we report BAGV and BJV nucleic acids were detected in pools of *Cx. perexiguus*. BJV has been previously detected in pools of *Cx. perexiguus* in Israel; however, vertebrate hosts of BJV have not been identified [17].

Initially isolated from *Culex* mosquitoes in the Central African Republic in 1966 [65], BAGV has also been isolated from Senegal [66–68] and India [69] and was determined to be synonymous with Israel turkey meningoencephalomyelitis virus [70]. An outbreak of BAGV was responsible for high mortalities in pheasants and partridges in Spain in 2010 [71]. Although BAGV nucleic acid is typically found in pools of *Culex* mosquitoes, including those in the Univitattus complex [66–68], to our knowledge the virus has never before been identified in *Cx. perexiguus*. Laboratory experiments have been performed to test for vector competence for BAGV in *Ae. aegypti*, *Cx. bitaeniorhynchus*, and *Cx. quinquefasciatus* [72]. It was determined that *Cx. bitaeniorhynchus* was capable of transmitting the virus to suckling mice although virus was detected in the saliva of all three species [72]. In our survey, *Cx. bitaeniorhynchus* were only collected from WW, the natural habitat; and although *Cx. quinquefasciatus* were collected from all habitats, including AAZ, flavivirus was not detected from this species. It remains to be tested if *Cx. perexiguus* is a competent vector of BAGV.

We evaluated mosquito biodiversity and the presence of arboviruses in three habitats: a human-made peri-urban greenspace (AAZ), a peri-urban human-made wetland (RK), and an undisturbed natural habitat (WW). Mosquito species diversity was highest in the undisturbed natural habitat (WW, *H*′ = 1.36) and lowest in the man-made artificial habitat (AAZ, *H*′ = 0.46) (Table 3). In general, invertebrate species richness tends to decrease with increased levels of urbanization [4], although this is highly taxon-specific [73]. Although few studies have investigated the connection between mosquito biodiversity and urbanization, our data agree with the observations of others that species diversity decreases with increased urbanization/anthropization [6, 14, 15].

In parallel with this observation of reduced species alpha diversity, anthropization may lead to the creation of habitats which favor certain mosquito species. Previous studies have focused on the predominance of anthropophilic species in urban areas as a result of both host abundance and anthropogenic habitat creation [15, 73, 74]. In our study, the artificial brackish water habitat (RK) had the lowest species richness (*S* = 3) but relatively high evenness (*J*′ = 0.66) (Table 3). In this case, larval habitat was likely the most important autecological driver of mosquito biodiversity: the larvae of *Ae. caspius* are highly salt tolerant and the presence of treated wastewater is an ideal habitat for *Cx. quinquefasciatus* [55]. The case at the other artificial peri-urban green space, AAZ, is less clear. AAZ had high species richness (*S* = 7), although nearly 90% of the collection was *Cx. perexiguus* (lowest evenness, *J*′ = 0.23). *Cx. perexiguus* larvae utilize a wide range of stagnant water sources for their development [55] and adults feed mainly on birds but also on mammals [60, 75]. Thus, the abundant stagnant water sources and dense vertebrate population at the site provided suitable opportunities for *Cx. perexiguus* at AAZ. In contrast, the natural habitat (WW) had a high species richness (*S* = 6) and the highest species evenness (*J*′ = 0.76) (Table 3), further supporting the observed trend that urbanization may favor certain species by creating homogeneous ecological niches.

We investigated peri-urban habitats because these are thought to be key interfaces between humans and enzootic viruses where spillover is most likely to occur [11, 15]. In this study, mosquito-borne viruses were associated with the artificial greenspace of AAZ, and this site had the lowest mosquito alpha diversity and lowest species evenness. The relationship between urbanization and arbovirus activity is a complex one, and involves not only the vector population but also the susceptible host population [6, 9, 14, 15, 75–78]. In this case, AAZ had a robust vertebrate population representing many native and non-native species. Thus, anthropogenic habitat creation favored a single mosquito species, and the dense population of potential vertebrate hosts may have been optimal for increased presence of arboviruses at AAZ. However, no arboviruses were discovered in RK, where habitat alteration was related to a reduction in species diversity and species richness compared to the natural habitat, despite the presence of *Cx. perexiguus* and another known arbovirus vector, *Ae. caspius*. Future studies should expand arbovirus surveillance efforts in peri-urban sites, as they may allow arbovirus spillover opportunities.

## Conclusions

We performed a survey of the mosquito population at three sites in the UAE. We recorded the presence of 10 species, of which all were previously recorded in the Arabian Peninsula and five represent first confirmed reports from the UAE. We detected two viruses in pools of *Cx. perexiguus* at a peri-urban trap site in eastern UAE, one of which, BAGV, has been associated with avian mortality in Europe. As expected, the site of highest mosquito biodiversity was the protected natural site in WW, and biodiversity was lower in the human-made site in Al Ain where the viruses were identified. Anthropogenic landscaping may have favored the predominance of *Cx. perexiguus* at AAZ and RK, and the presence of the arboviruses at AAZ may be related to an abundance of susceptible hosts. The mosquito distribution in peri-urban sites was correlated with anthropogenic autecological drivers that favored certain mosquito species.

## Abbreviations

AAZ: Al Ain Zoo
BAGV: Bagaza virus
BJV: Barkedji virus
RK: Ras Al Khor Wildlife Sanctuary
UAE: United Arab Emirates
WW: National Park Wadi Wurayah
WNV: West Nile virus
